# Netrin as a Novel Biomarker and Its Therapeutic Implications in Diabetes Mellitus and Diabetes-Associated Complications

**DOI:** 10.1155/2018/8250521

**Published:** 2018-09-18

**Authors:** Ebrahim M. Yimer, Kaleab Alemayehu Zewdie, Hailemichael Zeru Hishe

**Affiliations:** Department of Pharmacology and Toxicology, College of Health Sciences, Mekelle University, Ethiopia

## Abstract

Diabetes is a multifactorial metabolic syndrome and is one of the shared long-lasting illnesses globally. It is linked to long-term microvascular and macrovascular complications that contribute to disability, compromised quality of life, and reduction in lifespan, which eventually leads to death. This disease is not only incurring significant economic burden but also adversely affects the patients, caregivers, communities, and the society at large. The interruption of diabetes progress and its complications is a primary focus of scientific communities. In spite of various diagnostic modalities for diabetes, there is a limited marker to investigate the risk and progress of its complications. Netrin has recently received more attention as a biomarker of diabetes and a broader range of long-term complication. Therefore, the impetus of this review is to exhaustively discuss the role of Netrin as a potential biomarker and its therapeutic implication in diabetes and diverse sets of microvascular and macrovascular complications of diabetes. It also discourses the possible mechanisms of Netrin for the said pharmacological effect for a better understanding of the development and progression of diabetes and its complications in relation to this protein. It enables protective measures to be applied at the subclinical stage and the responses to preventive or therapeutic measures to be scrutinized. Besides, it might also facilitate the appraisal of novel therapeutic options for diabetes and various complications through modifying the endogenous Netrin and provide surrogate endpoints for intervention.

## 1. Introduction

Diabetes mellitus (DM) is one of the globally shared enduring metabolic disorders designated by persistent elevation of plasma sugar level [1, 2]. It is commonly classified as type 1, type 2, gestational diabetes, and specific types of DM owing to other bases, of which type 2 diabetes is the commonest form. Diabetes has a multifaceted pathogenesis that occurs either due to impaired insulin secretion or due to development of insulin resistance at target tissues and/or wide-ranging destruction of pancreatic *β*-cells [1, 3].

### 1.1. Pathogenesis and Incidence of Diabetes and Related Complications

This unresponsiveness of insulin and pancreatic *β*-cell damage leads to the failure of insulin to normally regulating dietary metabolism, and the *β*-cell-associated modifications further induce the cellular signaling cascade. For instance, *β*-cell dysfunction initiates the stimulation of advanced glycation end products, diacylglycerol kinase pathway, oxidative stress, metabolic stress, and inflammation which in turn reduce the *β*-cell functioning, causing a sustained rise in blood glucose [3, 4]. Once chronic hyperglycemia happens, individuals living with diabetes are highly susceptible to various forms of both short- and long-term complications. Among the short-range diabetes complications, ketoacidosis, hyperosmolar hyperglycemic state, and comma are the commonly encountered problems. On the contrary, macrovascular and microvascular complications, including cardiovascular disorders, nephropathy, retinopathy, stroke, foot ulcer, and neuropathy, are associated with long-term diabetes complications that could end up in a substantial morbidity and mortality [3, 5–7]. This tendency of increased morbidity and mortality seen in diabetic patients is more pronounced because of its insidious onset and late recognition, especially in resource-limited nations including Africa [8].

The incidence of diabetes is promptly growing universally as a result of aging population, urbanization, and other associated lifestyle modifications [9]. In 2017, it was estimated that there are about 451 million (age 18–99 years) individuals with diabetes globally and this figure is expected to increase to 693 million by 2045, of which 90% of them had T2DM [10, 11]. The figure of individuals with diabetes is projected to rise to 592 million by 2035, of which 7.7% of the general societies denotes the global productive age groups [12]. According to the review conducted in 2016, the worldwide approximation of diabetes in adult population aged 20–79 was half billion, with an African country prevalence of 3.2% and an estimation of 0.8–3.5 million individuals living with diabetes in Ethiopia. The prevalence is expected to reach 28 million by 2030 in Africa [13].

The risk of mortality among people with DM is about twice that of individuals of the same age without diabetes. Diabetes is ranked the 7^th^ leading cause of fatality and prominent complications comprising of lower limb amputations, visual impairment, end-stage renal disease (ESRD), birth complications, sexual dysfunction, heart disease, and stroke [14]. The average prevalence of acute kidney injury (AKI) worldwide was estimated as high as 30% in adults [15]. Diabetic retinopathy (DR) also affects approximately 80% of DM patients [16]. According to Tracey et al., the occurrence of diabetes complications in Ireland is ranged 6.5–25.2% for retinopathy, 3.2–32.0% for neuropathy, and 2.5–5.2% for nephropathy [17].

A study conducted in northern Africa also showed that the prevalence of retinopathy and nephropathy is 41.5% and 46.3%, respectively, in hospitalized patients and the prevalence of diabetic neuropathy also reached 60% in inpatient hospital clinics of Egypt. Diabetic neuropathy in Sudan was also reported to be as high as 31.5% in inpatient clinics and 36.7% in outpatient clinics [9]. The long-lasting complications of diabetes in Ethiopia are also estimated with the prevalence of 35%, 25%, and 15% of neuropathy, retinopathy, and nephropathy, respectively. Similarly, diabetic foot ulcer and impotency (25% and 44%, respectively) are highly prevalent in diabetic patients [18].

Although various studies suggested that the progression of diabetes can be postponed or prevented with earlier initiation of current treatment protocols, the prediction and early identification of diabetic complications are challenging. For instance, diabetic retinopathy still has no definitive diagnostic means [4], and for other complications having diagnostic tools, their sensitivity and/or specificity is too poor [19, 20]. The efficacy of diabetic management to delay progression of diabetes mellitus would be improved if they could be implemented during the initial phases of the disease and targeted at individuals with a maximal possibility of benefitting from the therapeutic intervention. To achieve this goal, identifying new biomarkers for predicting individuals at high risk of diabetes and its complications has, therefore, become a priority for targeting preventive measures efficiently [21, 22].

Given the alarming incidence of diabetes and associated complications in the global community and the adverse influence of diabetes complications on the quality of life of the patients and their caregivers, a new approach for the prompt diagnosis and innovation of an effective and safe treatment option is required. Thus, this review is intended to discuss the role on Netrin as a novel biomarker so as to detect earlier diabetic complications or predictor of diabetic risks and as a possible therapeutic goal so as to propose safe and effective antidiabetic agents.

### 1.2. Overview of Netrin and Its Receptors

**Figure 1:**
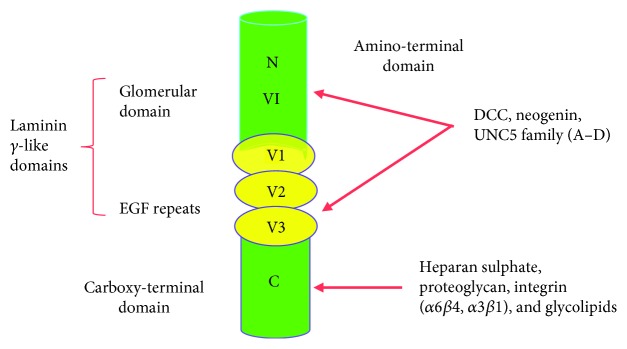
Primary designation of Netrin protein expression and their main receptors. EGF: epidermal growth factor; UNC5: uncoordinated 5; DCC: deleted in colorectal cancer.

Netrin is a family of extracellular, laminin-related proteins [23], comprising of Netrin-1, Netrin-3, and Netrin-4, and dual glycosylphosphatidylinositol-attached membrane peptides (Netrin G1 and G2) have been described in humankind [24]. Netrin-1 was among the first to be recognized and well-characterized as a member of Netrin. This peptide is comprised of nearly six hundred residues of an amino-terminal domain VI, followed by 3 laminin-type epidermal growth factor replications (V-1, V-2, and V-3) and a carboxy-terminal domain [25, 26] as illustrated in Figure 1. The biological roles of Netrin is mediated through two well-recognized receptor families, namely, the deleted receptors in colorectal cancer (DCC) and the uncoordinated 5 (UNC5) receptors [27, 28], but added receptors such as CD146, also termed melanoma cell adhesion molecule and Down syndrome cell adhesion molecule, might also be involved [29, 30].

The DCC subfamily comprises DCC and neogenin, via which Netrin-1 generally mediates axon attraction [27]. The ectodomain of DCC is composed of four immunoglobulin- (Ig-) like domains and six fibronectin type III (FNIII) domains. The DCC FNIII repeats mediate interactions with Netrin-1 through its LN-LE 1–3 region. That region, when added as an Fc-fusion protein, is sufficient to mimic the axon outgrowth activity of full-length Netrin-1 [31]. Intracellularly, DCC does not encrypt any noticeable catalytic domain but consists of 3 exceedingly conserved arrays, namely, the P1–3 motifs. DCC intermediates chemoattractant to Netrin-1–4 and also contributes to chemorepellent signaling responses [29]. Neogenin, which is part of the family of DCC, shares about 50% amino acid uniqueness to DCC and interacts with Netrin-1 and -3 but also binds with structurally distinctive repulsive guidance molecule (RGM), an alternate ligand that does not own to the subset of Netrin [29]. A knockdown analysis in zebrafish supported the role of neogenin in mediating axonal attraction to Netrin, but this function has not been established in mammals yet, where it has mostly been studied as an adhesive factor and a putative guidance receptor for RGM [31]. In addition to their function of guiding axonal development, both DCC and neogenin control cell-cell linkage and tissue organization via interfaces with the secreted Netrins [29].

Another family, UNC5, is mainly responsible for axon repulsion, and it consists of the UNC5 A–D receptor complex. Of these, UNC5B, expressed during early blood vessel formation, is the utmost essential and is implicated in Netrin-1-controlled new blood vessel formation (angiogenesis) [25, 27]. The extracellular portion of UNC5 comprises two Ig domains, followed by 2 thrombospondin type I segments. Its intracellular area comprises a ZU5 domain of unknown purpose, a DCC-interacting spot, and a death domain that is linked with apoptotic signaling [25, 29].

Together with DCC, UNC5 family members are termed dependence receptors, because of the dependence of cell survival on the presence of the Netrin-1 ligand, and the absence of these receptors is known to induce apoptosis and the interaction of Netrin-1 to DCC exerts a significant downregulation of tumorigenesis and angiogenesis [7, 23]. Hence, Netrin-1 regulates cell migration, cell-cell interactions, and cell-extracellular matrix adhesion at the time of embryonic development of numerous tissues, including the nervous system and the vasculature, pulmonary, pancreatic, and muscular systems, as well as the mammary gland [32]. There are also a number of evidences that support the involvement of Netrin in several pathologies including cancer [33], cardiovascular disorders [34], and neurological conditions [35]. Thus, this review gives emphasis on the role of Netrin in diabetes and mainly on its micro- and macrovascular complications.

## 2. Netrin and Diabetes Mellitus

Netrin is classically recognized as a neural guidance cue that has been involved in various tissues including pancreas development. Because Netrin's tissue regenerative, angiogenic, and inflammatory suppression properties have been reported in different studies, its effects in the islet of *β*-cells and glucose homeostasis in various preclinical and clinical studies conducted so far are discussed.

The expression and function of Netrin-1 in the regulation of neuronal cell migration using pancreatic fetal and adult rats revealed a momentary expression of Netrin-1 mRNA in the fetal pancreas and post-ligation in the adult pancreatic duct. Netrin-1 expression was detected together in endocrine as well as exocrine cells. A histochemical analysis also showed that, of the two well-recognized Netrin receptors, neogenin was highly expressed in the pancreas that indicates Netrin-1 involvement in pancreatic morphogenesis, tissue remodeling, and islet-cell migration as well as rejuvenation [36]. A model of developing epithelium using human embryonic pancreatic cells evidenced that Netrin expression, particularly Netrin-4, is significantly expressed in pancreatic ductal cells as well as the vascular endothelium. This assists that epithelial cell connection via integrin *α*2*β*1 and *α*3*β*1 and Netrin-4 recognition through these integrins stimulates insulin and glucagon genetic expression. Fetal pancreatic cell linkage to Netrin-4 also causes a noticeable downregulation of cyclins and upregulation of negative regulators of the cell cycle that might act as a prodifferentiation signal for pancreatic cells [37]. The effects of Netrin-1 and Netrin-4 and their receptors in *β*-cell action, apoptosis, and proliferation were further evaluated. A downregulation of caspase-3 was detected once cells were bared to exogenous Netrin-1 and -4 in hyperglycemic states. The reduction in caspase-3 cleavage, in turn, was associated with the diminution of neogenin and UNC5-A receptors. On the other hand, neogenin and UNC5 receptors showed to bring apoptosis in the absence of Netrin while they prevent apoptosis upon interaction with Netrin [38]. This finding points out the role of Netrin in the prosurvival of *β*-cells. On the contrary, the genetic expression of adipose tissue from mice nourished a regular diet or high-fat diet (HFD) were compared and a substantial rise in adipose expression of Netrin-1 and UNC5B from HFD-fed obese mice was noted as compared to lean chow-fed mice. The high expression of Netrin-1 supports defective adipose tissue migration and retention which, in turn, enhance the progression of chronic inflammation, insulin resistance, and metabolic dysfunction [39].

Similarly, after thirty days' continuous administration Netrin-1 to HFD-/STZ-induced diabetic mice, the stimulatory effect of Netrin-1 on insulin release from *β*-cells was noted via promotion of Ca^2+^ influx and the cAMP signaling pathway, which is alike with neuronal axon growth/guidance cone response. A hypoglycemic asset of Netrin-1 was also verified, which is possibly attributed to enhancing *β*-cell function, presented as amplifying the levels of insulin and pre-proinsulin mRNA expression. Besides, intensified islet vascularization and diminished islet macrophage infiltration were detected (Nicol, Hong, & Spitzer, 2011; [27]).

A recent clinical study by Jung et al. claimed that Netrin-1 may be a new biomarker for early detection of impaired fasting glucose (IFG) or T2DM. Briefly, they found a significant increment of serum Netrin-1 level in subjects with IFG or T2DM compared to the control group; serum Netrin-1 levels had a significant positive correlation with fasting glucose, HbA1c, HOMA-IR, AST, and ALT. Also, a statistically inverse correlation was found between Netrin-1 and HDL cholesterol and eGFR levels. On top of that, serum Netrin-1 was independently associated with the presence of IFG or T2DM [40]. On the contrary, Liu et al. conducted a clinical study on 56 human subjects, where 30 subjects who had new-onset type 2 diabetes were allocated for the treatment group while the remaining were assigned for the control group to assess the extent of Netrin-1 in diabetic patients. They found that the level of Netrin-1 in diabetic patients was meaningfully reduced than that of healthy controls. Additionally, the extent of Netrin-1 was found to be inversely related with homeostasis model evaluation of insulin resistance and plasma glucose (fasting and post-meal), fasting insulin, triglyceride, and hemoglobin A1c levels [4]. So the above two clinical studies showed a contradictory finding regarding Netrin-1 level and DM which requires further investigation to determine the actual relationship.

## 3. Netrin and Diabetic Complications

### 3.1. Netrin and Retinopathy

Diabetic retinopathy (DR) is one of the commonest microvascular complications in hyperglycemic patients that can occur when the tiny blood vessels in the retina become impaired. These vessels come to be tumefied and leaked or they might be protected and blood precluded from passing through it [41]. DR is characterized by specific loss of pericytes, which leads to an augmented blood vessel permeability, and the development of new blood vessels, which is also called retinal neovascularization [42, 43]. DR is considered a common cause of visual impairment mainly through macula edema and vitreous hemorrhage [42]. The overaccumulation of plasma glucose in diabetic patients leads to the damage of tiny blood vessels and augments the level of inflammatory mediator, prostaglandin E2, by activating the NF*κ*B factor in the retina [44]. All these alterations ultimately lead to loss of vision and permanent blindness in diabetic patients unless prompt interventions are carried out.

Relative decrease in oxygen supply and ischemia are the basic reasons for the pathological growth of neovascularization. In the case of diabetic subjects, long-term hyperglycemia can trigger inadequate blood supply that eventually leads to blood-retina barrier breakdown, high vascular permeability, and avascularity. Thus, numerous angiogenic related cytokines, such as hypoxic-inducible factors (HIFs), vascular endothelial growth factor (VEGF), and erythropoietin are overexpressed to raise blood flow of the ischemic tissue, to increase vascular permeability, and to maintain the perfusion pressure in the tissue [45]. Retinal neovascularization might also occur in diverse ocular diseases other than diabetic retinopathy such as retinopathy of prematurity and secondary neovascular glaucoma [46].

Although DR is a major type of diabetic complication and the main cause of blindness in diabetic patients, till now there is no known biomarker that suggests the prompt alarms of retinopathy as well as its severity in DM patients. Currently, a number of evidences indicated that neural guidance cues and their binding sites, such as ephrin, Netrin, and semaphorins, function as angiogenic regulators. It has been reported that Netrin-1 could induce a proangiogenic phenotype in endothelial cells and stimulate developmental and therapeutic neovascularization [46, 47]. Other than its role in neovascularization, it is also involved in guiding the exit of retinal ganglion cell axons from the eye and the extension of these axons into the optic nerve. It also has a central role in optic fissure closure in embryonic eye development and attracts dorsal commissural interneurons when it interacts to the DCC receptor [42, 45]. A single subconjunctival administration of Netrin-1 in diabetic mice also displayed an expressive shortening in the rate of corneal epithelial wound healing than that of the diabetic control [48].

Fortunately, in recent years, various experimental and human trials are suggesting the role of Netrin in diabetes or chemical-induced retinopathy as an innovative marker and potential therapeutic target, which is summarized in Table 1. According to the findings of most of these studies, alteration of the body's Netrin level possibly is considered as a future biological protein to detect retinopathy as early as possible and to determine its severity, which in turn, assists for new drug discovery for this troublesome disease condition.

### 3.2. Netrin and Nephropathy

Diabetic nephropathy is a tubular disease of the renal system primary due to alteration of tubular epithelial cells and is an important factor in the development of progressive kidney diseases of either acute or chronic kidney damage. Inflammatory response from tubular epithelial cells can affect different parts of the kidney, including vasculature and glomerular mesangial cells, via inflammatory mediators such as cytokines, chemokines, and prostanoid metabolites. These mediators will bring hyperfiltration, matrix expansion, apoptosis, and vasodilation and further increase the production of their own and other mediators of cellular damage [44].

Acute kidney injury (AKI) is a common form of nephropathy, which is defined as a quick (within 48 h) drop-down of renal function resulting in failure to conserve body electrolyte, acid-base, and fluid homoeostasis. Diabetes becomes the principal cause of nephropathy, and various animal and human studies suggest that acute and chronic kidney diseases (CKD) are associated with inflammation in which inflammatory mediators play a major role in tissue damage of both forms of nephropathy [19, 50]. Cells have a defensive mechanism that often is activated in parallel with the inflammatory response to counteract the damaging effects of innate immune cells. These cytoprotective molecules include anti-inflammatory cytokines, neuronal guidance cues, Netrins, adenosine, hemeoxygenase, and others. However, inadequate response or downregulation of these counteracting pathways may exacerbate inflammatory response and tissue injury [50].

Currently, the diagnosis of renal derangement depends on a reduction in glomerular filtration rate (GFR) and a rise in serum creatinine (Scr) with or without oliguria, which is described by two classification systems: the Acute Kidney Injury Network (AKIN) and the RIFLE (Risk, Injury, Failure, Loss, and End-stage) criteria of kidney disease. Although these diagnostic modalities are considered good predictors of nephropathy, they are neither sensitive nor specific mainly in the setting of early detection of AKI. Furthermore, alterations of Scr and blood urea nitrogen (BUN) concentrations chiefly reflect functional changes in filtration capacity instead of factual injury markers [19, 20]. In order to address such difficulties, diverse novel biomarkers, particularly Netrin protein, are currently receiving more attention as a new marker to detect AKI and CKD with enhanced specificity and sensitivity.

**Figure 2:**
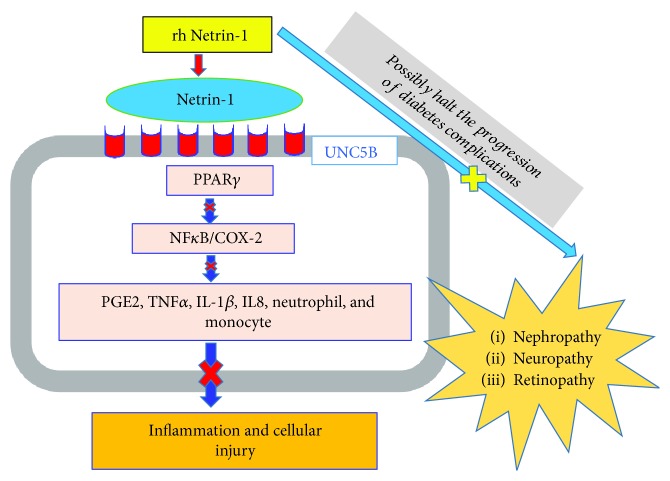
Schematic illustration of the possible molecular mechanism of anti-inflammatory actions of recombinant human Netrin-1 mediated through UNC5B and via commonest inflammatory signaling pathways and its potential role of affecting the progression of diabetic microvascular complications. PGE2: prostaglandin E2; TNF: tumor necrosis factor; COX: cyclooxygenase; PPAR: peroxisome proliferator-activated receptor; rhNetrin-1: recombinant human Netrin-1.

Netrin-1, the axon-guidance molecule has recently become an investigational protein in modulating inflammation, apoptosis, and many other pathological alterations in renal tubular epithelial cells. For instance, Netrin-1 anti-inflammatory actions were mediated through diabetes-induced COX-2 expression and PGE2 production. This suppressive effect of COX-2 was expedited through inhibition of NF*κ*B activation. These inflammatory suppressant actions of Netrin-1 were proposed to modulate not only diabetic nephropathy but also the progression of various microvascular diabetic complications [44, 51, 52] (Figure 2).

In addition, Netrin-1-mediated reduction in albuminuria occurs by enhancing the uptake of albumin by proximal tubular epithelial cells through the activation of PI3k and ERK pathways. In various animal and human studies, it has been reported that Netrin-1 was highly secreted later on both acute and chronic kidney diseases [20, 53–55]. Comparable with the depletion of serum Netrin-1 level, UNC5B mRNA as well as Netrin-1 were established to be substantially diminished in diabetic kidney, while albuminuria/proteinuria was found to be overexpressed in mice with deleted UNC5B/Netrin-1 in kidney and administration of recombinant Netrin-1 considerably reduced diabetes-induced albuminuria and repressed interstitial and glomerular injuries [56].

The serum Netrin-1 concentration in microalbuminuric diabetic patients was also markedly elevated compared to normoalbuminuric diabetic patients and the control group. The increment of plasma Netrin-1 was found to be positively and negatively correlated with albuminuria and estimated GFR, respectively [57], which suggest the likelihood of glomerular damage. Numerous studies have been conducted to examine the role of Netrin in different animal models of nephropathy and human trials (Table 2). Based on the findings of most of the studies, plasma and/or urinary Netrin-1 level alterations were noticed which are adversely associated to albuminuria and estimated GFR of various animals and human studies, which at least partly explained the glomerular damage in diabetic/chemical-induced nephropathy. Other than its role as a biomarker, this protein might also be considered as a potential therapeutic target to develop novel agents to overcome AKI, CKD, and/or renal fibrosis [58].

### 3.3. Diabetic Neuropathy and Netrin

Diabetic neuropathy (DN) is among the commonest microvascular complications of DM. Population-based studies have indicated that more than half of the patients with either type 1 or type 2 diabetes develop DN, and as much as 30% of those manifestations are painful [59]. Neuropathic complications can be due to autonomic or sensory dysfunctions which affect either the periphery, gastrointestinal, genitourinary, or all other systems. Sensory complications include numbness, paresthesia, and tingling sensation in the extremities, leading to an intensification of serious foot ulceration in diabetics that might lead to amputation. Meanwhile, autonomic complications including postural hypotension, sexual dysfunction, bladder dysfunction, and gastrointestinal distress might also occur [60].

The elevation of blood sugar plays a crucial role in the progression and development of diabetic neuropathy. One of its mechanisms to cause DN is neural degeneration through increased oxidative stress. The metabolic abnormality and oxidative stress disorders cause very rapid changes in glial cells [2]. Mechanical allodynia might be produced due to the abnormal development of myelinated afferent fibers in the spinal dorsal horn which is associated with postherpetic neuralgia as well as peripheral nerve damage. Spinal cord injury also elicits central sprouting of A*β* afferents and neuropathic hyperalgesia [61].

It has been demonstrated that the earlier intensive glucose control will reduce the risk of neuropathic complications and will be practicing earlier metabolic regulation by using novel biomarkers, also showing longstanding effects on this clinical outcome. For instance, the importance of ephrins, slits, semaphorins, and netrins for the composition of the nervous system is nowadays well-comprehended, particularly their capacity to regulate defined axon targeting. From these four common axonal guidance family proteins, Netrin-1 has a durable chemoattractive capacity to enrich axonal extension and is highly expressed in the adult nervous system particularly after nerve damage [24, 67].

According to Dun and Parkinson, Netrin-1 plays a crucial role in upholding Schwann cell multiplication, peripheral nerve regeneration, and migration. So to stimulate the restoration of damaged peripheral nerves and serviceable recovery, targeting the Netrin-1 signaling pathway would be a novel therapeutic strategy [24, 68]. Lee et al. also assessed the expression of Netrin receptors in Schwann cells using various analytical methods and revealed that UNCB5B is required for Netrin-1-induced proliferation of RT4 schwannoma cells. UNC5B is the sole receptor expressed in adult primary Schwann cells. Netrin-1 and UNCB5B are found to be highly expressed in the injured sciatic nerve while Netrin-1-induced Schwann cell proliferation was antagonized by the specific inhibition of UNCB5B expression with RNAi. These data also suggest that Netrin-1 could be an endogenous trophic factor for Schwann cells in the injured peripheral nerves [68].

Similarly, the functional role of Netrin-1 on mechanical allodynia and sprouting of myelinated afferent fibers in resiniferatoxin- (RTX-) induced postherpetic neuralgia (PHN) and neuropathic pain were assessed by Wu et al. According to this study, Netrin-1 expression has been increased after spinal cord injury and inflammatory cells in the wound region, which in turn increased the levels of Netrin mRNA expression after this injury. On the other hand, RTX treatment meaningfully amplified Netrin-1 expression in the spinal dorsal horn and is highly expressed in human neuroblastoma cells, SH-SY5Y. The effect of the transient receptor potential vanilloid (TRPV1) agonist and antagonist on the Netrin-1 expression was also assessed, and it was found that RTX dramatically increased the level of Netrin-1 expression which was antagonized by the TRPV1 antagonist, capsazepine [61].

### 3.4. Netrin and Cardiovascular Diseases

Among macrovascular complications of diabetes, cardiovascular disorders are the most significant sequelae. Most diabetic patients will die due to various cardiovascular diseases (CVDs) such as coronary artery disease (CAD), cerebrovascular disorder, peripheral vascular illness, and stroke. Of these CVDs, majority of mortality is attributable to CAD, which is mostly due to atherosclerosis [2, 60].

In the past few years, Netrin-1 has been explored to play an essential role in atherosclerosis, ischemia/reperfusion injury, and angiogenesis through involving a cardioprotective peptide, though its defined role in these disorders was protective or deleterious and has been the area of controversy. The credentials of DCC and UNC5-binding sites on cell types other than neurons have supported the notion that Netrin-1 could have extra utilities beyond the CNS. Over the past decade, it has become apparent that Netrin-1 has enrolment in several biological reactions, extending from angiogenesis to inflammatory process, making it a striking prospective novel pharmacologic target for CVDs [34, 69, 70]. In the latest years, numerous animal- and human-based studies have been conducted to confirm the role of Netrin in various CVDs as an investigational biomarker and possible therapeutic target by modifying associated pathophysiologic mechanisms (Table 3). Based on the findings from these studies, Netrin might be considered as a future potential biomarker for prompt identification of diabetes-related CVDs and other related causes.

#### 3.4.1. Role of Netrin-1 in Angiogenesis

Angiogenesis is the common physiologic process in which new blood vessels are generated from an existing vessel. Although it is a homeostatic development that principally arises during the embryogenic process, angiogenesis also occurs in adults in the course of the ovarian cycle and normal physiological restoration process [71].

Blood vessels and nerves often follow matching paths, proposing to utilize distant targets as a shared signal that brings vascularization and innervations [34]. The vascular endothelium is essential in controlling vascular smooth muscle tone through intensifying the production of an endogenous vasodilator, nitric oxide. Vascular endothelium dysfunction (VED) is a crucial and influencing aspect in the occurrence of diabetes-brought vascular complications. Diabetes-induced decrement of L-arginine accessibility via the amplified arginase activity can cause nitric oxide synthase (NOS) uncoupling, excessive generation of reactive oxygen species (ROS), reduced NO levels, and VED [7, 34].

Netrin-1 has shown to enhance proliferation, initiate cell relocation, and stimulate linkage of endothelial and vascular smooth muscles with a specific effect parallel to vascular endothelial and platelet-derived growth factors. The mechanism by which Netrin-1 stimulates angiogenesis has also been revealed: Netrin-1-mediated initiation of angiogenesis is NO-facilitated, and stimulation of NO entails extracellular signal-regulated kinase (ERK) 1/2 and DCC, which is instigated following the activation of DCC receptors in endothelial cells. In contrast, the introduction of NO scavengers or an antibody to DCC inhibited Netrin-1-induced angiogenesis in endothelial cells [34, 72].

#### 3.4.2. Expression of Netrin-1 in Atherosclerosis

Atherosclerosis is a condition in which fatty materials are accumulated in the wall of the artery and eventually block the artery. It is characterized by progressive inflammation, accumulation of lipids, and fibrosis [73, 74]. The arterial inflammatory response is initiated by the subendothelial preservation of plasma LDL and promoted by oxidative alteration of this lipoprotein, which elicits an inflow of monocytes. Contrasting to other inflammatory states, atherosclerotic inflammation does not readily resolve and cholesterol-loaded macrophages persist in the arterial wall. These macrophages are also called the prime source of foam cells that cause extension of the plaque through enrollment of further leukocytes and vascular smooth muscle cells and contribute substantially to plaque instability [70].

Different studies have been investigated that Netrin protein prevents the migration of monocytes, neutrophils, and lymphocytes via the receptor of UNC5B (Table 3). Netrin-1 is predominantly expressed by macrophage foam cells, which is established in both in vitro and in vivo models, as well as in atherosclerotic lesions. Indeed, these studies revealed that Netrin-1 expressed by foam cells controlled the cellular constituents of atheroma. Netrin-1 inactivated macrophage migration and supported chemoattraction of coronary artery smooth muscles. Netrin-1 also strongly reduces leukocyte recruitment into the vascular wall in atherosclerosis, and lack or inhibition of Netrin-1 by proatherogenic factors has shown to increase leukocyte adhesion to the endothelium [34, 74, 75].

#### 3.4.3. Role of Netrin-1 in Hypertension

Hypertension (HTN) is a common form of cardiovascular disorder that is defined as persistent elevated arterial blood pressure (BP) (i.e., BP > 140/90) [73]. The effects of poorly controlled diabetes can lead to the kidney to develop structural and functional abnormalities, which include hyperfiltration with glomerular hypertension, renal hypertrophy, increased glomerular basement membrane thickness, tubular atrophy, and interstitial fibrosis. These events subsequently prime to the formation of proteinuria and aggravated systemic hypertension. Unfortunately, blocking the renin-angiotensin system by presently available therapies provides only limited protection against the progression of these disease conditions [55]. The existence of hypertension as a comorbid condition in diabetic patients also upsurges the chance of getting diverse microvascular complications like diabetic nephropathy and macrovascular complications such as stroke. Many endogenous molecules are under investigation to utilize them as early detection of these conditions and as a therapeutic target, of which the role of Netrin in HTN is given more emphasis as summarized in Table 3.

#### 3.4.4. The Effect of Netrin in Ischemic Heart Disease

Ischemic heart disease (IHD) is defined as a lack of oxygen and inadequate or no blood flow to the myocardium resulting from the narrowing of the coronary artery or obstruction. IHD may present as an acute coronary syndrome (ACS), which includes unstable angina and non–ST-segment elevation or ST-segment elevation myocardial infarction (MI), chronic stable exertional angina, ischemia without symptoms, or ischemia due to coronary artery vasospasm [73].

Reperfusion treatment of damaged myocardial tissue is the ultimate means for decreasing infarct size as well as improving patient outcome particularly in patients with ST-segment elevation myocardial infarction. Despite the restoration of coronary blood flow, this can paradoxically persuade further myocardial injury indicating reperfusion treatment as a “double-edged sword.” Reperfusion injury is an intricate spectacle mediated by numerous factors, including oxidative stress, intracellular calcium buildup, prompt restoration of acidity, and inflammatory response, and comprises a partly stimulation of the so-called mitochondrial permeability transition opening [34, 76].

Exogenous supplementation of Netrin-1 has shown a cardioprotective action against ischemia/reperfusion (I/R) injury through an increase in NO level, which is dependent on the DCC/ERK1/NOS/DCC feedforward signaling cascade. Netrin-1 also exhibited an improvement of MI in a diabetic animal model and abolishes I/R-induced cardiac mitochondrial dysfunction via NO-dependent attenuation of NADPH oxidase action and retortion of NOS. Additionally, Netrin-1 treatment has been shown to diminish autophagy, which occurs in a coronary ligation model of MI [7, 69].

#### 3.4.5. Role of Netrin-1 in Ischemic Stroke

Ischemic strokes are caused either by local thrombus formation or by the occurrence of emboli, which bring about an occlusion of a cerebral artery. Atherosclerosis, particularly of the cerebral vasculature, is a causal element in most circumstances of ischemic stroke, despite that 30% is cryptogenic. Emboli can arise either from intra- or extracranial arteries (including the aortic arch) or, as in some conditions heart is involved. A cardiogenic embolism is presumed to have occurred if the patient has concomitant atrial fibrillation, valvular heart disease, or any other condition of the heart that can lead to clot formation [67, 73, 77]. There are naturally occurring genetic modifications in synaptic plasticity-associated genetic material that may affect both stroke development and poor retrieval of functionality after stroke. Netrin-1, together with its ligand, NGL-1, promotes neurite outgrowth, controls synapse formation, and stabilizes excitatory versus inhibitory responses. In particular, this protein stimulates thalamocortical axonal outgrowth, induces and maintains excitatory synapse formation, and contributes to subdendritic division in the cortical and hippocampal areas. Additionally, some research output suggested that Netrin-1 is implicated in immune response, which is supposed to be an important element of ischemic stroke progression. This peptide is a guidance cue possibly enrolled in immune cell communications and trafficking and has a vital role in N-methyl-D-aspartate receptor stimulation, which elicits neuronal loss in the brain by modifying inflammation.

In addition, it has been proposed that Netrin-1 may inhibit leukocyte chemotaxis in microglia [77]. Hence, this protein might have many more clinical implications in various CNS disorders that demand further and in-depth evaluation.

Another study also demonstrated that Netrin-1 and its receptors, DCC and UNC5H2, were overexpressed in the infarct/peri-infract zone of an ischemic adult brain. The level of UNC5H2 was markedly elevated in neurons in the ipsilateral VPN at 8 and 14 days after middle cerebral artery occlusion, which was temporally and spatially linked to neuronal apoptosis, while the expression of DCC was only lightly detected [67]. This implies that contrasting in primary brain ischemia, UNC5H2, instead of DCC, was primarily involved in secondary neuronal death.

## 4. Conclusion and Future Perspectives

Although diabetes mellitus and its related complications have been diagnosed and managed in different diagnostic criteria and drug groups, the incidence of morbidity and mortality related with DM has increased alarmingly. For solving such problems, early identification and treatment of DM and its micro- and macrovascular complication are the ideal way of management. Netrin, the laminin-related protein which regulates cell migration, cell-cell interactions, and cell-extracellular matrix adhesion during the embryonic development of multiple tissues, including the nervous system, vasculature, lung, pancreas, muscle, and mammary gland, is used as a novel biomarker and therapeutic modality for early identification of DM and related complications.

Different animal models and human subjects that were induced/have diabetes alone or along with various micro- and/or macrovascular complications showed that the level of Netrin is altered in various disease conditions. The level of Netrin in a diabetes model displayed an inconsistent expression in different clinical studies which require further investigations. Even though there is a variability of Netrin expression in different micro- and macrovascular complications, overall, the extent was highly increased earlier as compared to corresponding control groups. Hence, an in-depth understanding of such pathological changes should be sought so as to design this protein as a novel biomarker and potential therapeutic targets for the management and early detection of DM and related complications.

Furthermore, a comprehensive identification of its substrates is necessary for a better understanding of the signaling cascades of Netrin, which possibly helps to explain the intricate intracellular signaling networks in a wide range of situations and to achieve such therapeutic hope, and further analysis of the expression of Netrin in diabetes and each complication will shed light on the biological mechanisms and prospective therapeutic applications.

## Figures and Tables

**Table 1:** The expression of Netrin in retinopathy.

Model	Methods and interventions	Treatment outcomes	References
Streptozotocin-induced diabetic rats (Sprague-Dawley rats).	After diabetes has been induced, rats were randomly divided into group 1 (diabetic eyes without treatment), group 2 (diabetic eyes with PBS treatment), 3^rd^ group (diabetic eyes with Netrin-1 (5.0 *μ*g/mL) received group), and 4^th^ group (diabetic eyes with Netrin-1 (5.0 *μ*g/mL) administered group).	The Netrin-1-receiving group was found to suppress and reverse retinal neovascularization significantly at a concentration of 5 *μ*g/mL compared to control and lowest concentration for the Netrin-1-administered group.	[43]
		While a 0.1 *μ*g/mL Netrin-1 displayed a meaningful raise in the no. of new retinal blood vessels, after six weeks' administration compared to diabetic controls	

Patients who have diabetic retinopathy (DR)	A total of 18 diabetic patients were included, of which 10 of them were patients having DR and 8 patients were without DR.	The levels of Netrin-1 and VEGF in the vitreous of patients having DR were expressively higher than those in the controls.	[45]
Oxygen-induced retinopathy (OIR) mouse models	Vitreous liquid samples were collected from the eyes of both groups using the pars plana vitrectomy technique.	Netrin-1 was primarily expressed in GCL and INL of the mouse retina.	
	Adequate blood samples were also collected.	Both Netrin-1 and VEGF were substantially upregulated in OIR mice.	

Type 2 diabetes patients and streptozotocin- (STZ-) induced diabetes mouse model.	Diabetic patients with DME and control patients without DMF were enrolled +	A substantial elevation of vitreous truncated Netrin-1 by 8 folds in patients with DME	[42]
	Mice received either STZ or sodium citrate buffer as a control group.	A significant augmentation of retinal edema was detected in DMF patients compared to the control.	
	After 8 weeks STZ administration, retinal vascular permeability rises by greater than two-fold as compared to the control group.	Truncated Netrin-1 expression was also significantly amplified in the STZ-treated mice.	
		An elevation of collagenase matrix metalloprotease 9 (MMP-9) was noted to have the capability of cleaving Netrin-1 into the laminin (VI-V) fragment.	

Animal model of oxygen-brought retinopathy (OIR) in C57BL/6J mice	Mice were placed in an oxygen chamber and exposed to less concentrated oxygen for 5 days and returned to room air to induce retinal neovascularization.	Asymmetrical neovascularization and fluorescein outflow were detected around the unperfused parts in the hypoxic cluster.	[46]
	Control mice were exposed only to room air for the same period of time.	The hypoxic cluster showed distended neovascular nuclei into the vitreous humor than the corresponding controls.	
	Reverse transcriptase PCR and Western blot analyses were used to examine retinal Netrin-1 mRNA and protein expression.	Netrin-1 mRNA levels were substantially increased in mouse retina of the hypoxic group compared to control.	
		Similarly, the extent of Netrin-1 protein in hypoxic than normoxic mice was highly expressed.	

Animal model of oxygen-induced retinopathy (OIR) in C57BL/6J mice	Mice were exposed to oxygen (75 ± 2%) for 5 days and then returned to normal air to induce retinal neovascularization.	Excluding UNC5A, Netrin-1 receptor subtypes UNC5B, UNC5C, UNC5D, DCC, and neogenin altogether were expressively amplified in the retinas of OIR mice.	[47]
	Reverse transcriptase PCR and Western blot were used to assess the expression of Netrin-1 receptor subtypes in the mouse retinas.	OIR mice treated with recombinant UNC5B shRNA displayed an intense reduction in neovascular extension into the interior restrictive membrane.	

Streptozotocin- (STZ-) induced diabetes rats (adult male Sprague-Dawley rats)	Rats were assigned randomly to a diabetic group (DM) and a control group (C), each group was containing of 10 rats.	Diabetic rats exhibited typical signs of diabetes.	[49]
	A single dose of STZ was administered to the diabetic group, and the control group received just a citrate buffer.	Cataract was detected in the DM group at 3 months after administration of STZ.	
		Both Netrin-1 mRNA and protein expression were notably augmented in the retina of DM rats compared to the control group.	

PBS: phosphate-buffered saline; DR: proliferative diabetic retinopathy; GCL: ganglion cell layer; INL: the inner nucleic layer; DME: diabetic macular edema; OIR: oxygen-induced retinopathy; STZ: streptozotocin; PCR: polymerase chain reaction; DCC: deleted colorectal cancer; VEGF: vascular endothelial growth factor.

**Table 2:** The extent of Netrin expression in nephropathy.

Model	Methods and interventions	Main findings	References
STZ-induced diabetes in proximal tubular epithelial UNC5B knockout mice and heterozygous UNC5B knockout mice (DBA/2J mice) and wild-type (WT) mice	After two months of diabetes induction, twenty-four hours' urine was collected	Both WT as well as heterozygous diabetic mice showed significant albuminuria compared to the control group.	[56]
	The animals were then sacrificed, and renal tissues were further processed for histological examination.	Urinary albumin excretion was more prominent in mice with proximal tubule particular deletion of UNC5B.	
		Substantial upregulation of Netrin-1 in proximal tubules in the heterozygous diabetic group was noted.	
		Recombinant Netrin-1 supplementation also considerably decreased in diabetes-brought albuminuria and repressed interstitial and glomerular injuries.	

Cross-sectional study of human subjects (obese patients and normal controls)	An overall of sixty-two nonalbuminuric and normotensive obese individuals (with and without insulin insensitivity) and sixty-four normal controls were involved in the study.	Obese subjects exhibited substantially greater Netrin-1 urinary excretion than the control groups.	[28]
		The level of Netrin-1 and creatinine in urine was also meaningfully amplified in obese patients with insulin resistance than those without insulin resistance.	
	Samples from urine and blood were collected to analyze the level of creatinine, albumin, and Netrin-1.		
		But no significant differences were detected between these subjects for the occurrence of microalbuminuria.	

Animal models of (5/6 nephrectomized (Nx) and sham-operated rats) chronic renal failure	Animals were randomly assigned into three groups (*n* = 10 rats/group): sham-operated rats received the control adenovirus; 5/6 Nx rats received with the control adenovirus (empty vector); and 5/6 Nx rats were treated with recombinant adenovirus (Ad-Netrin-1).	The 5/6 Nx groups showed markedly reduced Netrin-1 level compared with the sham-operated group	[58]
		The Ad-Netrin-1-received group displayed markedly increased Netrin-1 expression compared with the 5/6 Nx control group.	
		Despite that 5/6 Nx rats showed initial elevation, Ad-Netrin-1 treatment exhibited a remarkable reduction in Scr and BUN levels.	
		The effect of Netrin-1 to attenuate the progression of renal dysfunction might be via inhibiting EndoMT in 5/6 Nx rats.	

Observational studies on human subjects having AKI, non-AKI, and control subjects	Patients who were admitted to the ICU were divided into (a) patients who had AKI (49 subjects) and (b) non-AKI (101) patients and (c) fifty subjects as the control group.	The level of Scr began to rise after 24 h of admission while the extent of Netrin-1 has amplified expressively within the 1^st^ h and persistently elevated up to 48 h among AKI patients.	[20]
	For each patient, urine and blood samples were collected and assessed.	In contrast, Netrin-1 expression in patients who did not develop AKI stayed the baseline at different time intervals.	
		Continual elevation of urinary KIM-1 and Netrin-1 was noticed in septic AKI patients compared to non-AKI and control groups.	

Prospective case control study (on newborns with a diagnosis of perinatal asphyxia (PA))	An overall of 41 newborns identified with PA were enrolled in this study.	The levels of urinary NGAL, Netrin-1, NHE3, and IL-18 on the 1^st^ day were intensely elevated in patients compared to controls.	[54]
	Of which 15 patients with AKI while 26 without AKI and 20 healthy subjects as the control group.	Despite the extent that NGAL and IL-18 were intensified in AKI patients, the levels of Netrin-1 and NHE3 were comparable compared to patients without AKI.	
	Urine samples were collected on days 1 and 4 after birth from patients with PA and the control group.	For the samples taken on postnatal day 4, only NGAL levels were knowingly raised in AKI compared to non-AKI patients.	

Cross-sectional study (on patients who experienced orthotropic liver transplantation (OLT))	Sixty-three patients who underwent OLT were involved.	The urinary levels of Netrin-1, semaphorin 3A, and NGAL were amplified meaningfully and peaked at 2 h while Scr was raised after 48 h of post-transplantation in patients with AKI compared to matched non-AKI individuals.	[53]
	Among which individuals who had preexisting renal failure were excluded and patients that undertook OLT and had intact renal function were finally recruited.	The prognostic power of Netrin-1, as verified by the AUC for diagnosis of AKI at 2, 6, and 24 h post-transplantation, was 0.66, 0.57, and 0.59, respectively.	
	Preoperative samples (urine + blood) were obtained from individual patients at various time intervals.	The AUC for diagnosis of AKI was 0.63 and 0.65 for semaphorin 3A and NGAL at 2 h, respectively.	

Case-control study (on diabetes and nondiabetes subjects)	In this study, sixty diabetes (30 patients with microalbuminuria and 30 subjects without microalbuminuria) and fifty-six healthy volunteers were enrolled.	Plasma Netrin-1 level was suggestively higher in diabetes compared to the nondiabetic group whereas the level was comparable between the group without diabetes and nonalbuminuric diabetic patients.	[57]
	The plasma samples were collected after fasting (8–12 h), and urinary albumin level was assessed.	The level of plasma Netrin-1 in microalbuminuric diabetic individuals was meaningfully increased compared with the group without diabetes and the nonalbuminuric diabetic group.	
	Plasma and urinary creatinine, HbA1c, glucose, and cholesterol expressions were also quantified.	The higher Netrin-1 expression was positively correlated with the duration of diabetes, HbA1c, and microalbuminuria/creatinine ratio while it was inversely correlated with the level GFR (*P* < 0.033).	

STZ-induced diabetic mouse model (to assess renal proximal tubular epithelial- (RPTE-) cell expression of Netrin)	Diabetes was induced for both Netrin-1 transgenic mice and wild-type (WT) mice using STZ while the control group received vehicle.	The chicken Netrin-1 transgene was prominently expressed in transgenic mouse RPTE cells while minimal endogenous Netrin-1 expression was noticed in WT and transgenic mouse kidney.	[44]
	The level of Netrin-1, glucose, renal function, albumin, inflammation, and body weight were assessed at different points of time.	Endogenous Netrin-1 was overexpressed in WT mice with no change in transgenic mice at 36^th^ weeks of diabetic induction.	
		Diabetes-induced PGE2 production and urinary excretion were noticeably inhibited in Netrin-1 transgenic mice.	
		This PGE2 production suppressant effect of Netrin-1 was established through suppression of NF*κ*B-mediated COX-2 in RPTE cells.	
		Netrin-1 also amplified albumin uptake by RPTE cells via the PI3 kinase and ERK signaling without affecting glucose uptake.	
		Besides, the *in vitro studies revealed that hyperglycemia hampered the expression of Netrin-1 in RTPE cells.*	

STZ-induced diabetes and DOCA salt-induced hypertension model (in Sprague-Dawley rats and C57BL/6 mice)	Diabetes was induced for both rats and mice; control groups were given saline, and the test animals were implanted with palmitic acid blanks	Urinary Netrin-1 excretion was expressively higher in diabetic rats at 4 and 10 weeks after inducing DM as compared to control.	[55]
	After rats were anesthetized, the kidney was removed and a 2-month timed-release pellet of DOCA was embedded. The water consumption of DOCA-treated rats was subsequently followed for 1 month. Blood pressures were assessed weekly and after 4 weeks of DOCA and 10 weeks of STZ, respectively.	Urinary Netrin-1 level was also suggestively raised from the hypertensive group at 4 weeks as compared to controls.	
		A substantial elevation of albuminuria was noted in diabetic rats (on weeks four and ten) as compared to controls.	
		Similar to diabetic model in rats, early Netrin-1 excretion also augmented in diabetic mice and the highest expression was associated with disease severity.	

A single-center, cross-sectional study (diabetes with and without albuminuria)	A total of eighty-eight diabetes subjects (of which 40 had no albuminuria, 38 had microalbuminuria, and 9 had macroalbuminuria) and 42 nondiabetic individuals without CKD as control were enrolled.	Type 2 normoalbuminuric diabetes had higher Netrin-1 excretion as compared to control than matching type 1 DM patients.	[62]
		Urinary Netrin-1 levels were substantially elevated in diabetic subjects with normoalbuminuria compared to controls, proposing early tubular damage.	
		Netrin-1 expression was also elevated in late stages of nephropathy in both micro- and macroalbuminuric patients as compared to controls; however, the level of Netrin-1 between these two groups of albuminuria was insignificant.	
		A noticeable positive correlation was noted between urine Netrin-1 and occurrence of CV disorders, albumin/creatinine ratio, plasma creatinine, and HbA1c, while there was a negative association with urinary creatinine.	

Peritubular capillary (PTC) loss and hypoxia in 5/6 nephrectomized (Nx) in rat models	Male sex rats (Sprague-Dawley) were randomly allocated into 3 groups: (a) sham-operated rats received control adenovirus; (b) 5/6 Nx rats received control adenovirus; and (c) 5/6 Nx rats were administered recombinant adenovirus-facilitated Netrin-1 (Ad-Netrin-1) gene.	The Ad-Netrin-1-received 5/6 Nx group exhibited a marked elevation of Netrin-1 expression compared to the 5/6 Nx group, though still lower than the sham-operated group.	[63]
	Blood urea nitrogen, serum creatinine, and 24-h urinary albumin excretion extents were quantified.	Ad-Netrin-1 administration induced a significant intensification in renal PTC density, accompanied by a substantial reduction in HIF-1*α* levels.	
	Histopathological alterations in kidney tissues were evaluated. The expression of Netrin-1 and hypoxia inducible factor-1*α* (HIF-1*α*) was also assessed.	Ad-Netrin-1 administration also attenuated PTC injury, diminished tissue hypoxia, and restored kidney function, which improve renal pathological alteration and interstitial fibrosis in 5/6 Nx rats than the 5/6 Nx control group.	
		Besides, a 24-h urinary albumin concentration was expressively decreased in the Ad-Netrin-1-received 5/6 Nx group, compared with the 5/6 Nx control group.	

Renal ischemia-reperfusion (RIR) induced AKI and CKD in C57BL/6J mice and age-paralleled Netrin-1 transgenic mice	Animal were grouped into treatment (administered recombinant IL-6 (10 ng) and Netrin-1 (250 ng/mL)) and control groups.	Netrin-1 transgenic mouse kidney function improved more quickly compared with wild-type.	[51]
	Animals were sacrificed after surgery, and renal tissue was taken and processed for protein isolation.	Upregulation of tubular Netrin-1 displayed a remarkable suppression of tubular atrophy and glomerular sclerosis.	
	Cells were then treated with IL-6, Netrin-1, or a combination of both IL-6 and Netrin-1 for 24 h.	The RIR interstitial fibrogenesis and capillary injury were inhibited by upregulation of Netrin-1, and expressions were also suppressed in Netrin-1 transgenic mice.	
		IL-6-boosted hypoxic fibrotic response in mouse RPTE cells was notably suppressed by Netrin-1.	

Prospective study on subjects having renal injury (allograft, ischemic AKI, AKI associated with sepsis, and radiocontrast- and drug-induced AKI) and healthy individuals	A total of 63 patients (22 subjects underwent a renal allograft, eleven with ischemic AKI, thirteen with AKI related with sepsis, 9 with radiocontrast-brought AKI, and 8 with drug-induced AKI) who had kidney injuries and 10 healthy controls were included in this observational study.	The initial urinary Netrin-1 level at 2 h after surgery revealed a very high concentration in renal transplant patients compared to the control.	[64]
	Evaluation of urinary Netrin-1 level was carried out by sandwich enzyme-linked immunosorbent assay.	These patients were monitored till day 18, and Netrin-1 levels kept decreasing as renal function improved and eventually disappeared from urine.	
		Netrin-1 levels were dramatically increased in urine from patients with ischemic ATN, sepsis, and radiocontrast-induced and drug-induced acute renal failure.	
		There was still high level of Netrin-1 found in the urine samples, suggesting the ongoing renal injury.	

Ischemia reperfusion in wild-type (WT) and RAG-1 knockout mice induced severe renal injury model	RAG1 knockout mice were subjected to about half an hour of ischemia after that reperfusion.	Both WT and RAG1 knockout mice developed severe IRI while the corresponding sham-operated groups revealed no renal injury.	[65]
	Netrin-1 was given to the test groups.	The expression of inflammatory mediators (neutrophil, and cytokine and chemokine) was significantly suppressed in WT as well as in RAG1 knockout mice, which received Netrin-1 whereas the matching groups which received vehicle displayed a substantial expression of these inflammatory mediators.	
	IFN*γ* or LPS induced a high COX-2 expression and PGE2 formation in the macrophage.	The level of COX-2 expression was notably upregulated after reperfusion whereas the level of COX-1 was not changed. Injection of Netrin-1 repressed the level of COX-2 in both WT and RAG1 knockout mice.	
	PGE2 receptor EP4 selective agonist (ONO-AE1-329) activated ischemic renal injury (IRI) and polymorphonuclear cell infiltration	Renal reperfusion-induced ischemia exhibited an increment of the formation of PGE2 and its renal excretion, which was remarkably suppressed in groups that received Netrin-1.	
		Netrin-1-injected mice also displayed significant reduction of IFN*γ* and LPS-induced COX-2 and PGE2 concentrations.	

Ischemia reperfusion damage of the renal tissue and cisplatin-brought kidney toxicity by tissue-specific UNC5B receptor knockout and half-deficient mice	Mice with UNC5B2/flox/GGT-cre and matched mice without cre (UNC5B2/flox) or WT mice were subjected to about half an hour of ischemia then reperfusion.	Both UNC5B2/flox/GGT-cre group and corresponding mice without -cre passed away within 1-day after subjected to ischemia while the WT mice were subsisted.	[23]
	UNC5B2/flox/GGT-cre and UNC5B2/flox mice were exposed to 22 minutes of ischemia then a 48 h of reperfusion. Renal function was evaluated by determining the extent of Scr at different intervals.	WT mice did not display any elevation of Scr levels with a slight form of ischemia, whereas mice with RPTE cell-specific UNC5B deletion exhibited a substantial intensification in Scr and blood urea nitrogen.	
		Majority of the mice died by 72 h, which indicated that the UNC5B receptor has a crucial value against ischemic RPTE cells.	

Chemical-induced nephrotoxicity in mice and case control study in patients with AKI.	Renal ischemia-reperfusion and cisplatin, lipopolysaccharide (LPS), and folate were given for various groups of C57BL/6J mice to induce AKI.	A significant upregulation of Netrin-1 (by 47-fold) in renal epithelial cells after ischemia reperfusion injury was noticed.	[66]
	Urine is collected at different periods of time, and kidney function (BUN and Scr) and Netrin-1 levels were assessed.	On the contrary, the Scr levels were considerably increased only after 6 h and peaked at 24 h after reperfusion.	
		The urinary expression of Netrin-1 was notably amplified in the cisplatin-received group by 10 and 30 folds within 3 and 6 h, respectively, after administration while the Scr level started to rise in the middle of 24 and 48 h post-administration.	
		In the folate-brought nephrotoxicity group, urinary Netrin-1 excretion was noticed within 3 h after injection before substantial alteration on Scr was noted and the Netrin-1 level persisted for 48 h.	
		Urinary Netrin-1 excretion was also elevated (by 60 folds) in LPS-brought kidney dysfunction within 1 h and peaked at 6 h after administration.	
		Urinary Netrin-1 levels were elevated in AKI subjects while 4 patients presented a lower, but still noticeable expression compared with control.	

OLT: orthotropic liver transplantation; EndoMT: endothelial-to-mesenchymal transition; NGAL: neutrophil gelatinase-associated lipocalin; PTC: peritubular capillary; HIF-1*α*: hypoxia-inducible factor-1*α*; KIM-1: kidney injury molecule-1; NHE3: Na^+^/H^+^ exchanger isoform 3; PGE2: prostaglandin E2; COX-2: cyclooxygenase 2; LPS: lipopolysaccharide; BUN: blood urea nitrogen; Scr: serum creatinine; IFN: interferon; AKI: acute kidney injury; CKD: chronic kidney disease; DM: diabetes mellitus; RPTE cell: renal proximal tubular epithelial cell,

**Table 3:** The levels of Netrin in cardiovascular (CV) disorders.

CV disorders	Model, method, and interventions	Main findings	References
Coronary artery disease (CAD) and atherosclerosis	A cross-sectional study was conducted in eighteen patients with CAD who underwent elective CABG were included as a test group while fourteen patients who underwent valvular surgery were included as the control group.	Macrophage infiltration was increased in EAT of CAD patients.	[75]
	All patients had significant proximal left anterior descending (LAD) artery stenosis.	Expression of Netrin-1, UNC5B, and cytokines related to M1-macrophage subtype (IL-12, IL-18) in EAT were amplified in CAD patients.	
	After sample collection, immunohistochemical staining and real-time PCR were undertaken to determine the expression of Netrin and other entities.	Netrin-1 and UNC5B expressions were found to be linked with macrophage intrusion and polarization in EAT.	
	A cross-sectional study was also carried out in patients with CAD.	Expression of Netrin-1 and its receptors in the circulating monocytes and whole blood are unaltered in coronary atherosclerosis.	[74]
	The left internal thoracic artery (LITA) samples serving as controls were obtained during coronary artery bypass surgery due to symptomatic CAD.	Netrin-1 was downregulated while UNC5B receptors became overexpressed in atherosclerotic plaques compared to normal controls.	
	After assessing necessary patient information, whole blood and monocyte collections were performed.	Netrin-1 and NEO1 correlated negatively and positively with macrophage-specific and SMC signature.	
	By collecting fresh arterial tissue samples, RNA is isolated and levels of arterial and whole blood samples of Netrin were assessed.	It was also noted that Netrin-1 was colocalized with CD68, which is indicative of cells of monocytic origin in the atherosclerotic plaques.	
	An in vivo animal study was conducted in low-density lipoprotein receptor (LDLR) knockout mice.	hNetrin-1 delivery exhibited substantial inhibition of lipid deposition and blood flow velocity.	[78]
	The animals received a high cholesterol diet (HCD).	It also showed significantly diminished aortic structural changes associated with atherosclerosis.	
	Human (h) Netrin-1 (cDNA by adeno-associated virus type 8) was administered to test animals and compared with controls of neomycin resistance (Neo) gene delivery/HCD.	hNetrin-1 delivery was also associated with lower inflammation as compared to the control group.	

Hypertension	The DOCA-salt induced hypertension and STZ-induced diabetes in male Sprague-Dawley rats and male c57bl/6 mice were used. After induction of hypertension with administration of DOCA. Blood pressures were measured weekly using the tail-cuff method.	DOCA rats gained less weight and displayed hyperphagia, polydipsia, and polyuria	[55]
	After 4 weeks of DOCA and after 10 weeks of STZ, rats were anesthetized and a terminal blood sample was collected from the abdominal aorta and level of Netrin-1 was assessed.	The expression of Netrin-1 was substantially elevated in hypertensive rats.	
		Induction of Netrin-1 protein was localized in renal proximal tubular epithelial cells in both diabetic and hypertensive models.	
		Increased albumin excretion in urine was seen in both diabetic and hypertensive rats.	
	Case control study was conducted in 72 women, of which 44 patients were with preeclampsia (PE) and the rest were normal pregnant mothers as a control.	Mean serum Netrin-1 level was remarkably higher in the study group compared to the control group.	[79]
	The PE group was divided into two subgroups as mild PE (*n* = 25) and severe (*n* = 19).	Despite not being statistically significant, serum Netrin-1 expressions were found to be higher in the subgroup of severe than mild PE.	
	Blood samples were collected from each participant, and a random urine (midstream) sample was collected to assess the serum Netrin levels, and serum Netrin-1 and urinary protein levels were finally evaluated.	It was also noted that pregnant mothers with mild and severe preeclampsia have significant urinary protein excretion than corresponding controls.	

Myocardial infraction	Streptozotocin-induced diabetes in male C57BL/6J mice and male Sprague-Dawley rats.	Intramyocardial administration of Netrin-1-MSCs decreased collagen buildup and precludes cardiac hypertrophic remodeling in rats and diabetic mice.	[69]
	After the T2D mouse model was set up, left anterior descending coronary artery enduring ligation was done to bring MI.	Intramyocardial administration of Netrin-1-MSC promotes neovessel formation.	
	The grouped mice were instantly administered with saline (MI + saline group), MSCs (MI + MSC group), or Netrin-1-expressing MSCs (MI + Netrin-1-MSC group).	Upregulation of NO release was noted, which was shown to enhance the expression of Netrin-1 by binding to its receptor, DCC.	
	Mesenchymal stem cells (MSCs) are isolated from mice and rats and histomorphological and immunofluorescence assay was performed.		
	Protein and NO expression was then quantified by Western blotting methods.		
	Animal models of myocardial IR injury were done via cardiac transplantation-induced MI in male C57BL/6 mice.	The level of Netrin-1 was downregulated after myocardial IR injury.	[76]
	After transplantation, transplanted hearts were assessed using an isolated working heart apparatus after 8 h of ischemia and 24 h of reperfusion. Serum analysis of Netrin-1 and myocardial apoptosis were evaluated by taking tissue samples from cardiac isografts.	Netrin-1 ameliorated myocardial IR injury	
		Netrin-1 reduced cardiomyocyte apoptosis and leukocyte infiltration.	
		Netrin-1 generated alternatively activated macrophages through PPAR*γ* activation	
	Wild-type (WT) C57BL6/J mice were subjected to a 30 min coronary occlusion after adequate anesthesia.	Netrin-1 showed a marked attenuation of ischemia reperfusion- (I/R-) induced myocardial infarction in the tested group.	[80]
	After a 24 h reperfusion with vehicle (normal saline), Netrin-1, UO126 (MEK1/2 inhibitor), PTIO (nitric oxide/NO scavenger), Netrin-1/UO126, and Netrin-1/PTIO intraventricularly for test and control groups were administered.	Netrin-1 also exhibited to recover cardiac function after ischemia reperfusion.	
	At the end of each 10 or 30 min of reperfusion, the heart was isolated and the LV was immediately frozen in liquid nitrogen.	This cardioprotective effect of Netrin-1 was found via a DDC-dependent mechanism.	
	Then various peptides were assessed after taking blood samples using different modalities.	ERK1/2 and NO were required for Netrin-1-mediated cardioprotection activity.	
		In addition, Netrin-1 exhibited a significant moderation of mitochondrial superoxide production via DCC and ERK1/2.	
		Netrin-1 besides showed an attenuation of autophagy in post-MI remodeled heart.	

Ischemic stroke	Cerebral ischemia was induced in adult male C57BL/6J mice by craniotomy in the left distal middle cerebral artery (dMCA). The carotid arteries were then occluded bilaterally for 20 min and then released.	The level of Netrin-4 was amplified in the ischemic core and colocalized with blood vessels after ischemia.	[81]
	Exogenous Netrin was infused into the lateral ventricle after induction of ischemia.	Netrin-4 was also expressed in the astrocytic foot processes in the peri-infarct cortex.	
	Sham-operated mice underwent identical surgery except that the dMCA and the common carotid arteries were not occluded	Intracerebroventricular administration of Netrin-4 was shown to enhance angiogenesis.	
	After animals were sacrificed, brains were isolated and the tissue corresponding to the ischemic core, peri-infarct cortex, and homologous contralateral cortex was dissected various analysis.	Expression of the putative Netrin-4 receptor DCC, but not UNC5A or UNC5B, was overexpressed in the peri-infarct cortex after induction of stroke.	
	A prospective study was conducted in 127 patients with ischemic stroke (IS) and 128 normal subjects.	The distribution of genotypes for NTNG1 and rs628117 SNP in both study groups complied.	[77]
	Among IS patients involved, 28 had cardioembolic stroke, and 99 had large-vessel atherothromboembolic stroke.	The finding showed that rs628117^∗^G minor allele frequency was markedly higher in patients compared to controls.	
	Blood and DNA samples were taken and analyzed for the expression of Netrin G1 gene (NTNG1) and rs628117 single-nucleotide polymorphism (SNP).	The carrier of this allele was overexpressed significantly in patients than corresponding controls (83 vs. 62%). The variation in the carriage of the NTNG1 rs628117^∗^G allele between the patients and controls extended to 98%.	
		Thus, from this study, it is noted that *NTNG1* rs628117 SNP might be a possible risk factor for IS.	
	Renovascular hypertension was induced in male Sprague-Dawley rats by bilateral renal artery clipping, and 96 rats with stable HTN were chosen.	UNC5H2 expression was substantially overexpressed in NeuN-positive neurons in the ipsilateral VPN after MCAO.	[67]
	Systolic blood pressure was measured at baseline and weekly.	Exogenous Netrin-1 treatment showed significant improvement of neurological function after MCAO.	
	Focal infarction was induced in the right dorsolateral cerebral cortex by electrocoagulation of the distal middle cerebral artery (MCA) and right MCA to produce MCA occlusion (MCAO).	Exogenous Netrin-1 administration also displayed a remarkable increment and decrement of the number of neurons and apoptosis, respectively.	
	Rats with developed permanent MCAO were randomly picked to receive continuous intracerebroventricular infusions of either Netrin-1 or control.		
	Neurologic evaluation of cell phenotypes Netrin-1, DCC, or UNC5H2 was conducted.		

CHF (cardiac hypertrophy)	Pressure overload models (thoracic transverse aortic constriction (TAC)) and culture of neonatal rat cardiomyocytes were performed in wild-type C57BL/6J mice and male Sprague-Dawley neonatal rat pups.	Expression of Netrin-1 reduced in murine hearts following TAC.	[82]
	Test groups receive recombinant Netrin while the control received vehicles.	Netrin-1 exhibited marked suppression of the cardiac fetal gene expression.	
	Mice's in the TAC and sham groups were then sacrificed and their hearts quickly excised, and mRNA levels of Netrin protein and other markers were analyzed.	Netrin-1 was also shown to attenuate the development of cardiac hypertrophy and heart failure.	
		Besides, Netrin-1 impeded the pressure overload mediated via MEK-ERK1/2 and JNK1/2 signaling pathways.	

MSC: mesenchymal stem cells; HCD: high cholesterol diet; TAC: thoracic transverse aortic constriction; PCR: polymerase chain reaction; MCA: middle cerebral artery; MCAO: middle cerebral artery occlusion; MSC: mesenchymal stem cells; NO: nitric oxide; EAT: epicardial adipose tissue; DCC: deleted in colorectal cancer; UNC5: uncoordinated-5; PPAR*γ*: peroxisome proliferator-activated receptor *γ*; CABG: coronary artery bypass grafting; IS: ischemic stroke; NOX4: NADPH oxidase isoform 4.

## Abbreviations

ACS: Acute coronary syndrome
AKI: Acute kidney injury
BUN: Blood urea nitrogen
CABG: Coronary artery bypass grafting
CAD: Coronary artery disease
CKD: Chronic kidney diseases
COX: Cyclooxygenase
CVD: Cardiovascular disease
DCC: Deleted in colorectal cancer receptors
DM: Diabetes mellitus
DN: Diabetic neuropathy
DR: Diabetic retinopathy
EndoMT: Endothelial-to-mesenchymal transition
ESRD: End-stage renal disease
HbA1c: Hemoglobin A1c
HCD: High cholesterol diet
HIF-1*α*: Hypoxia inducible factor-1*α*
I/R: Ischemia/reperfusion
IHD: Ischemic heart disease
IS: Ischemic stroke
KIM-1: Kidney injury molecule-1
MI: Myocardial infarction
NGAL: Neutrophil gelatinase-associated lipocalin
NHE3: Na^+^/H^+^ exchanger isoform 3
NOS: Nitric oxide synthase
OIR: Oxygen-induced retinopathy
OLT: Orthotropic liver transplantation
PPAR*γ*: Peroxisome proliferator-activated receptor *γ*
ROS: Reactive oxygen species
UNC5: Uncoordinated 5 receptors
VEGF: Vascular endothelial growth factor.
